# Long‐Term Low‐Dose of Nitrate Administration Improves Antioxidant Defence System in Insulin‐Sensitive Tissues of Type 2 Diabetic Rats

**DOI:** 10.1002/edm2.70186

**Published:** 2026-02-26

**Authors:** Fatemeh Ghorbani, Majid Shokri, Sajad Jeddi, Asghar Ghasemi

**Affiliations:** ^1^ Endocrine Physiology Research Center, Research Institute for Endocrine Molecular Biology, Research Institute for Endocrine Sciences Shahid Beheshti University of Medical Sciences Tehran Iran

**Keywords:** insulin resistance, nitrate, nitric oxide, oxidative stress, type 2 diabetes

## Abstract

**Introduction:**

Type 2 diabetes (T2D) is characterised by increased oxidative stress, which contributes to insulin resistance in insulin‐sensitive tissues. The objective of this study was to determine the antioxidative effects of long‐term nitrate administration in the liver, soleus muscle (SM) and epididymal adipose tissue (eAT) of male rats with T2D.

**Method:**

Rats were divided into four groups (*n* = 7): Control, Control+Nitrate (C + N), T2D and T2D + Nitrate (T2D + N). T2D was induced using a high‐fat diet followed by a low dose of streptozotocin (30 mg/kg). Nitrate (100 mg/L in drinking water) was administered for 6 months to the nitrate‐treated groups. Liver, SM and eAT were isolated, and tissue levels of catalase (CAT), total antioxidant capacity (TAC), reduced glutathione (GSH), malondialdehyde (MDA) and nitric oxide (NO) metabolites (NOx) were measured at the end of the study.

**Results:**

In the liver, nitrate increased CAT (216%, *p* < 0.001) and restored reduced TAC and GSH to normal values in rats with T2D. In the SM, nitrate decreased MDA (28%, *p* = 0.041) and restored reduced CAT to normal value in rats with T2D. In the eAT, nitrate increased CAT (72%, *p* = 0.046) and TAC (223%, *p* = 0.018) in rats with T2D. In addition, nitrate‐treated T2D rats had lower MDA (21.6%, *p* = 0.098) in the liver as well as higher TAC (104%, *p* = 0.064) in the SM and GSH (163%, *p* = 0.055) in the eAT; however, these changes were only marginally significant. Positive correlations were observed between NOx and CAT (*p* < 0.05 in SM and eAT), TAC (*p* < 0.05 in eAT) and GSH (*p* < 0.05 in liver, SM and eAT); furthermore, a marginally significant negative correlation was observed between NOx and MDA (*p* = 0.082 in liver and SM, *p* = 0.088 in eAT).

**Conclusion:**

Long‐term nitrate administration at low doses has a protective effect against oxidative stress in the liver, SM and eAT of rats with T2D.

## Graphical abstract

Long‐term nitrate administration at low doses has a protective effect against oxidative stress in the liver, soleus muscle (SM) and epididymal adipose tissue (eAT) of male rats with type 2 diabetes (T2D).

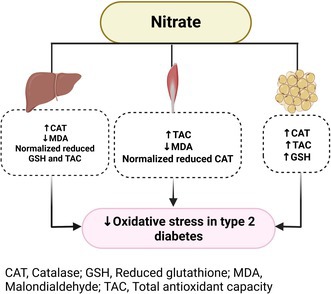

## 1 Introduction

The worldwide prevalence of type 2 diabetes (T2D) was 589 million in 2024 and is projected to reach 853 million by 2050, representing a 45% increase in the number of people living with T2D [1]. T2D is characterised by an increased oxidative stress [2], which contributes to impaired β‐cell function [3] and the development of insulin resistance in insulin‐sensitive tissues, including the liver [4], skeletal muscle [5] and adipose tissue [6]. Oxidative stress also contributes to the development of diabetes‐related complications, including cardiovascular disease, neuropathy, nephropathy and retinopathy [7], highlighting the need for a shift from solely glycemic‐based to a pathophysiology‐based approach [8] in managing T2D.

Experimental studies have shown that nitrate exerts beneficial metabolic effects in insulin‐sensitive tissues through multiple mechanisms. In adipose tissue, nitrate increases glucose uptake, reduces adipocyte size and stimulates the browning of white adipose tissue [9]. In skeletal muscle, nitrate increases the expression of glucose transporter 4 (GLUT4) mRNA and protein [10] as well as insulin‐induced Akt phosphorylation [11]. In the liver, nitrate decreases mitochondrial hydrogen peroxide and increases AMP‐activated protein kinase (AMPK) signalling [12, 13]. Additionally, recently, Li et al. established a paradigm shift in inorganic salt signalling biology that redefines nitrate as a direct ligand capable of exerting signalling effects in mammals, such as decreasing reactive oxygen species (ROS) and activating AMPK signalling independently of its reduction to nitrite and nitric oxide (NO) [14, 15].

Antioxidant effects of nitrate in T2D have been reported only in the heart and skin in the short term; nitrate (160 mg/L for 7 days [16] and 100 mg/L for 60 days [17]) decreased malondialdehyde (MDA) levels in the skin [16] and heart [17]. Favourable metabolic effects of nitrate are time‐dependent; for instance, long‐term (≥ 6 months) but not short‐term nitrate administration improves diabetes‐induced anaemia [18] and ovariectomy (OVX)‐induced osteoporosis [19]. The best of our knowledge, no study has addressed the antioxidant effects of nitrate in insulin‐sensitive tissues in T2D. Therefore, the objective of this study is to evaluate whether long‐term, low‐dose nitrate treatment (100 mg/L for 6 months) affects antioxidative parameters in the liver, soleus muscle (SM) and epididymal adipose tissue (eAT) of rats with T2D.

## 2 Materials and Methods

### 2.1 Animals and Ethics

According to the principle of the 3Rs (Replacement, Reduction and Refinement [20]), the number of rats used in this study was reduced by utilising tissues obtained from our previous project, which examined the effects of nitrate on carbohydrate metabolism in male rats with T2D [18]. All experimental procedures were approved by the ethics committee of the Research Institute for Endocrine Sciences, affiliated with the Shahid Beheshti University of Medical Sciences (IR.SBMU.ENDOCRINE.REC.1404.113).

### 2.2 Experimental Design

**FIGURE 1:**
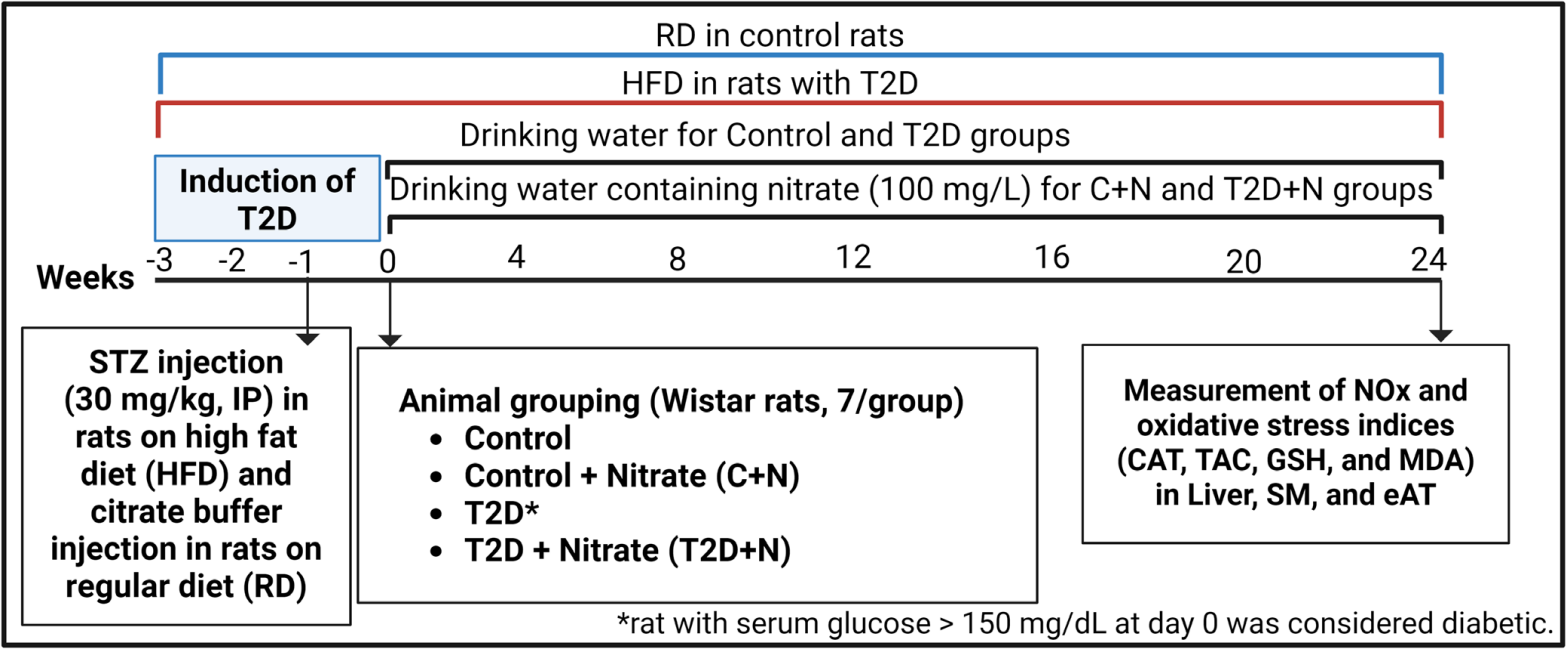
Experimental design of the study. CAT, catalase; eAT, epididymal adipose tissue; GSH, reduced glutathione; HFD, high fat diet; IP, intraperitoneal; MDA, malondialdehyde; NOx, nitric oxide metabolites; RD, regular diet; SM, soleus muscle; STZ, streptozotocin; T2D, type 2 diabetes; TAC, total antioxidant capacity.

As shown in Figure 1, 28 male rats were divided into 4 groups (*n* = 7/group): control, control+nitrate (C + N), T2D, and T2D + nitrate (T2D + N). T2D was induced using the combination of a high‐fat diet (HFD) and low‐dose streptozotocin (STZ, 30 mg/kg, intraperitoneal (IP), dissolved in citrate buffer, 0.1 M, pH 4.5), as described previously [21]. Rats in the nitrate groups received sodium nitrate at a concentration of 100 mg/L in their drinking water for 6 months, and untreated rats received only tap water. At month 6, samples of the liver, SM and eAT were isolated and tissue levels of NO metabolites (NOx) as well as CAT, TAC, GSH and MDA were measured based on the methods described previously [19]. In this study, protein concentration was measured using the Bradford method [22] and NOx as well as CAT, TAC, GSH and MDA levels are expressed as per mg protein.

### 2.3 Induction of Type 2 Diabetes

After receiving HFD for 21 days, STZ (30 mg/kg, IP) was administrated [21]. Seven days after STZ administration, rats with a fasting glucose concentration > 150 mg/dL were classified as type 2 diabetic rats. Control and diabetic rats consumed regular diet (RD) and HFD during the study, respectively. Details on preparing the HFD and STZ administration have been previously reported [21, 23].

### 2.4 Measurement of Tissues' Oxidative Stress Indices

At month 6, rats were anaesthetised by IP administration of sodium pentobarbital (60 mg/kg). After confirming deep anaesthesia, the tissue samples, including liver, SM and eAT, were collected and flash‐frozen in liquid nitrogen before being transferred to −80°C for further experiments. CAT, TAC, GSH and MDA in the liver, SM and eAT were measured at month 6. A portion of the tissues (100 mg) was homogenised in phosphate‐buffered saline (100 mM, pH 7.4, 1:5, weight per volume (w/v)) and centrifuged for 10 min at 10,000 g at 4°C. The supernatants from the homogenised tissues were then utilised to measure oxidative stress indices, which have been reported in detail elsewhere [19].

The CAT activity was measured using the Hadwan method [24]. In this method, 20 μL of supernatants were added to test tubes [containing 200 μL of H_2_O_2_ reagent (335 μL H_2_O_2_ 30% in 50 mL sodium–potassium phosphate buffer, 50 mM, pH 7.4)] and control tubes (containing 200 μL of distilled water). In addition, 20 μL of distilled water was added to the standard tube (containing 200 μL of H_2_O_2_ reagent). After incubating for 3 min at 37°C, we added 400 μL of a dichromate acetic acid reagent (60 mL glacial acetic acid and 20 mL of 5% potassium dichromate) to the tubes and incubated for 10 min at 99°C. This process converts dichromate to chromic acetate with H_2_O_2_, with the concentration of H_2_O_2_ directly proportional to the chromic acetate produced. CAT activity was calculated using the following formula: CAT activity (mU/min) = 2.303/(time of incubation) × Log [(absorbance of the standard tube)/(absorbance of the test tube—absorbance of the control tube)] × (total volume of reagents in the test tube)/(volume of the serum). Following cooling and centrifugation, the absorbance of 300 μL of the supernatant was read at 570 nm. CAT activity had 3.5% intra‐ and 4.1% inter‐assay coefficients of variation (CVs). CAT activity is presented as mU/mg protein/min in all studies tissues.

Tissue TAC was assessed utilising the Ferric Reducing Antioxidant Power (FRAP) assay [25]. In brief, 50 μL of the sample were added to tubes containing the FRAP reagent (contained 100 mL of acetate buffer (300 mM, pH = 3.6), 10 mL of tripyridyltriazine (10 mM in 40 mM HCl), and 10 mL of FeCl_3_ (20 mM in distilled water)) and incubated at 37°C for 10 min. Following incubation for 10 min, absorbance was assessed at 593 nm. This method involves the reduction of the ferric tripyridyltriazine (Fe^3+^‐TPTZ) complex to its ferrous form, which displays a pronounced blue colour at low pH. Ferrous sulfate solutions were used (range: 0–200 μM) to generate standard calibration curves for TAC levels. The intra‐assay and inter‐assay CVs for TAC were 1.1% and 1.3%, respectively. TAC levels are presented as nmol/mg protein in all studies tissues.

The level of GSH was quantified using the Sedlak and Lindsay method [26]. Briefly, 20 μL of the samples were added to tubes containing 200 μL of 5,5′‐dithiobis (2‐nitrobenzoic acid) (DTNB) and incubated at 37°C for 5 min. The absorbance was then read at 412 nm. The assay is based on the reaction of GSH with DTNB, commonly called Ellman's reagent that produces the TNB chromophore, which has a maximal absorbance at 412 nm. GSH levels are presented as nmol/mg protein in all studies tissues. The rate of formation of TNB, measured at 412 nm, is proportional to the concentration of GSH in the samples. Intra‐ and inter‐assay CVs for GSH were 2.4%, and 2.9%, respectively. GSH (range: 0–100 μM) was used to construct standard calibration curve for determining GSH levels in current study.

The level of MDA was quantified using the Satoh method [27]. In this method, 100 μL of the sample was added to tubes containing 500 μL of 20% trichloroacetic acid (TCA), and the tubes were allowed to stand at room temperature for 10 min. The samples were centrifuged for 10 min, and the supernatant was discarded. The resulting precipitate was washed with 0.05 M sulfuric acid. Next, 500 μL of sulfuric acid and 600 μL of 0.67% thiobarbituric acid (TBA) in 2 M sodium sulfate (w/v) were added to the precipitate, and the mixture was incubated at 99°C for 30 min. After cooling the tubes, 800 μL of n‐butanol was added to each tube, followed by vortexing and centrifugation for another 10 min. The absorbance of the resulting pink‐coloured complex was then measured at 530 nm. The intra‐assay and inter‐assay CVs for MDA were 4.2% and 4.7%, respectively. MDA levels are presented as nmol/mg protein in all studies tissues. Standard calibration was constructed using 1, 2, 3‐tetra ethoxy propane (range: 0–20 μM) for determining MDA.

### 2.5 Measurement of Tissue's NO Metabolites

The NOx concentration in all tissues was measured by the Griess method [28], with slight modification. In brief, insulin‐sensitive tissues were homogenised in phosphate‐buffered saline (pH 7.4) and centrifuged for 10 min at 10,000 g. For measuring NOx, nitrate was reduced to nitrite by adding vanadium trichloride (100 μL of VCl_3_, 8 mg/mL in 1 M HCl, the solutions passed through the membrane filter before used), and then 50 μL of both N‐(1‐naphthyl) ethylenediamine (0.1% in distilled water) and sulfanilamide (2% in 5% HCl). Samples were kept for 30 min at 37°C, and the absorbance was then read at 540 nm. Sodium nitrate (range: 0–100 μM) was used to generate a standard calibration curve for determining NOx levels. NOx concentration is presented as nmol/mg protein in all studies tissues. The intra‐assay and inter‐assay CVs for NOx in the liver, SM, and eAT were 2.2%, 3.5% and 1.8%, and were 4.0%, 3.2% and 2.5%, respectively.

### 2.6 Statistical Analyses

Statistical analyses were done using the GraphPad Prism software (Version 8). Data are presented as mean ± SEM. One‐way analysis of variance followed by the Bonferroni post hoc test was used to compare oxidative stress indices between groups. In addition, the correlation between NOx level and the tissue's oxidative stress indices was determined using Spearman correlation analysis. *p* values < 0.05 were considered statistically significant, and values between 0.05 and 0.10 were considered marginally significant.

## 3 Results

### 3.1 Effect of Nitrate on Oxidative Stress Indices in Liver

**FIGURE 2:**
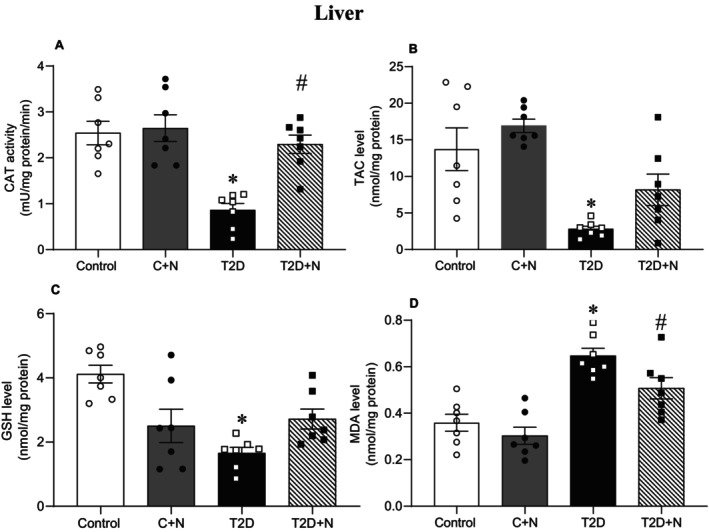
Effect of nitrate on oxidative stress indices in the rat liver. *, # Significant difference compared to control and non‐treated T2D groups, respectively; Values are mean ± SEM for 7 rats/group. C + N, control+nitrate; CAT, catalase; GSH, reduced glutathione; MDA, malondialdehyde; N, nitrate; T2D + N, type 2 diabetes + nitrate; TAC, total antioxidant capacity.

At month 6, compared to control rats, levels of CAT (66%, *p* < 0.001), TAC (80%, *p* = 0.002) and GSH (60%, *p* < 0.001) were lower, whereas MDA levels were higher (80%, *p* < 0.001) in the liver of rats with T2D. Nitrate increased CAT (216%, *p* < 0.001) and decreased MDA (21.6%, *p* = 0.098; although it was only marginally significant) in the liver of rats with T2D. Administration of nitrate restored TAC and GSH levels to near normal values in liver tissues of diabetic rats (Figure 2A–D).

### 3.2 Effect of Nitrate on Oxidative Stress Indices in Soleus Muscle

**FIGURE 3:**
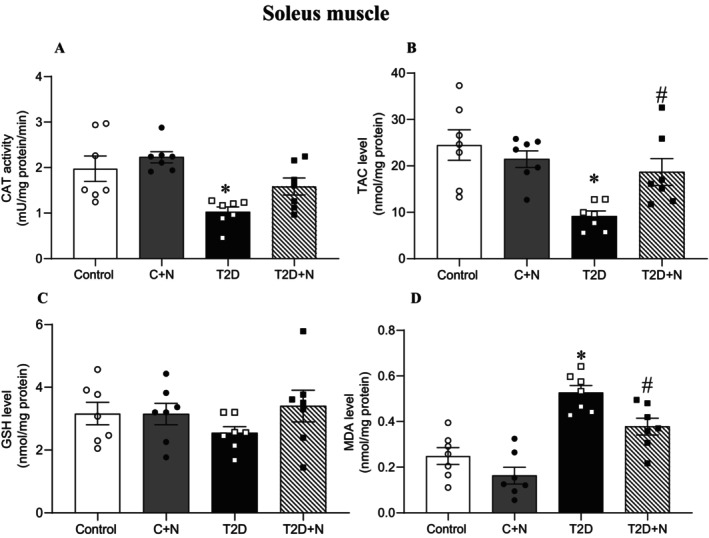
Effect of nitrate on oxidative stress indices in the rat soleus muscle. *, # Significant difference compared to control and non‐treated T2D groups, respectively; Values are mean ± SEM for 7 rats/group. C + N, control+nitrate; CAT, catalase; GSH, reduced glutathione; MDA, malondialdehyde; N, nitrate; T2D + N, Type 2 diabetes+nitrate; TAC, total antioxidant capacity.

At month 6, compared to control, levels of CAT (48%, *p* = 0.09, although it was only marginally significant) and TAC (63%, *p* = 0.001) were lower and MDA was higher in the SM (111%, *p* < 0.001) in the SM of rats with T2D. GSH was comparable between control and diabetic rats. Nitrate increased TAC levels (104%, *p* = 0.064; although it was only marginally significant) and decreased MDA levels (28%, *p* = 0.041) in the SM. Nitrate also restored CAT to near normal levels in the SM (Figure 3A–D).

### 3.3 Effect of Nitrate on Oxidative Stress Indices in Epididymal Adipose Tissue

**FIGURE 4:**
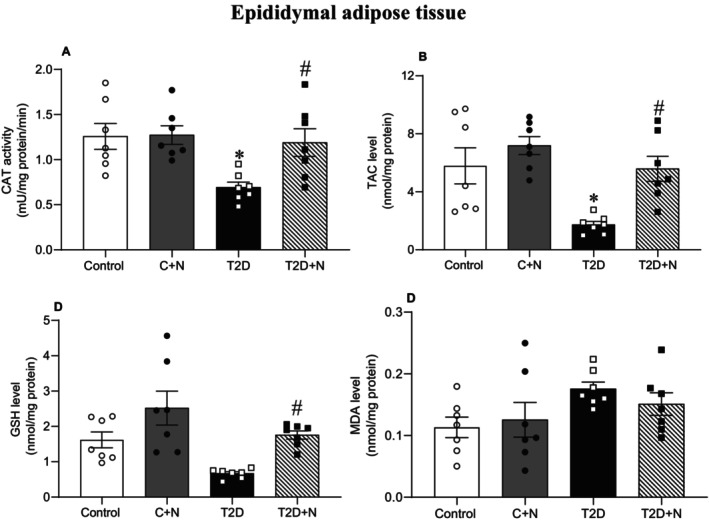
Effect of nitrate on oxidative stress indices in the rat adipose tissue. *, # Significant difference compared to control and non‐treated T2D groups, respectively; Values are mean ± SEM for 7 rats/group. C + N, Control+nitrate; CAT, catalase; GSH, reduced glutathione; MDA, malondialdehyde; N, nitrate; T2D + N, Type 2 diabetes+nitrate; TAC, total antioxidant capacity.

At month 6, compared to controls, levels of CAT (45%, *p* = 0.017) and TAC (70%, *p* = 0.011) were lower in the eAT of rats with T2D. MDA and GSH levels were comparable between control and diabetic rats. Nitrate increased CAT (72%, *p* = 0.046), TAC (223%, *p* = 0.018) and GSH (163%, *p* = 0.055, although it was only marginally significant) levels in the eAT of rats with T2D but did not affect MDA (Figure 4A–D).

### 3.4 Correlation Between Tissue NOx and Oxidative Stress Indices

The correlation between tissue NOx concentrations and oxidative stress indices is shown in Table 1. Among all rats (*n* = 28), NOx showed a positive correlation with CAT (although in liver, it was marginally significant), TAC (only in eAT) and GSH, and a marginally significant negative correlation with MDA.

**TABLE 1:** Correlation between nitric oxide metabolites (NOx) and oxidative stress indices in insulin‐sensitive tissues measured in all studied rats.

	Liver	SM	eAT
CAT	*r* = 0.366, *p* = 0.054	*r* = 0.536, *p* = 0.003	*r* = 0.508, *p* = 0.005
TAC	*r* = 0.192, *p* = 0.326	*r* = 0.192, *p* = 0.326	*r* = 0.487, *p* = 0.008
GSH	*r* = 0.462, *p* = 0.013	*r* = 0.462, *p* = 0.013	*r* = 0.600, *p* < 0.001
MDA	*r* = −0.333, *p* = 0.082	*r* = −0.333, *p* = 0.082	*r* = −0.327, *p* = 0.088

Abbreviations: CAT, catalase; eAT, epididymal adipose tissue; GSH, reduced glutathione; MDA, malondialdehyde; *n*, number; SM, soleus muscle; TAC, total antioxidant capacity.

## 4 Discussion

The main finding of this study is that long‐term (6 months) administration of a low dose (100 mg/L in drinking water) of nitrate has antioxidative effects in insulin‐sensitive tissues (liver, SM and eAT) of male rats with T2D. In addition, positive correlations were observed between tissue NOx and tissue CAT and GSH (in the liver, SM and eAT), TAC (in the eAT) and a negative correlation was observed between tissue NOx and tissue MDA in insulin‐sensitive tissues.

In current study, rats with T2D, showed increased oxidative stress in insulin‐sensitive tissues. In line with our results, decreased CAT [29], TAC [29] and GSH [29, 30], and increased MDA [16, 30, 31] have been observed in the liver [29, 30, 31], adipose tissue [31] and skeletal muscle [32, 33] of rats with T2D with different duration of T2D (28 [30], 30 [31] and 42 [29] days). Finding that for the first time was extended to 180 days by our study. These increased oxidative stress and decreased antioxidant capacity lead to lipid peroxidation, DNA damage and mitochondrial dysfunction, and contributes to the development of insulin resistance and T2D complications [34]. In addition, in current study, CAT activity and GSH level in the liver of control rats (set as a 100%) were higher than SM (77% and 76%) and eAT (49% and 39%), respectively; a finding in line with previous studies that liver antioxidative capacity is higher than other tissues, including skeletal muscle [35, 36, 37] and adipose tissue [35, 37, 38].

In our study, nitrate improved antioxidant activity in the liver, skeletal muscle and adipose tissue. Antioxidant effects on nitrate in T2D have been addressed only in the heart [17] and skin [16]; however, it has been shown that nitrite exerts antioxidant effects in insulin‐sensitive tissues (liver, SM and eAT) [39], pancreatic islets [3], and kidney [40] of male rats with T2D (Table 2). In the current study, nitrate administration increased CAT by 216% and restored the elevated MDA as well as reduced TAC and GSH to normal levels in the liver of rats with T2D. Similar to our results, decreased MDA levels following nitrate administration have been reported in the heart [17] and skin [16] of male rats with T2D. Additionally, nitrite (50 mg/L in drinking water for 2 months) exerts similar effects on CAT, MDA, TAC and GSH in rats with T2D [39]. Our results showed that in SM, nitrate decreased MDA by 28% and normalised reduced TAC, CAT and GSH in rats with T2D. Similar to these findings, the antioxidative effect of nitrite has been reported in SM, indicated by increased TAC, CAT and GSH and decreased MDA levels [39]. In the current study, in the eAT of rats with T2D, nitrate increased CAT by 72% and TAC by 223% and normalised the reduced GSH, but did not affect MDA. Partly in line with our results, it has been reported that nitrite increased TAC in eAT without affecting CAT, GSH or MDA. Notably, the shorter duration of nitrite treatment (63 days) may have been insufficient to affect CAT and GSH levels [39], which were observed following nitrate administration for 180 days in the current study.

**TABLE 2:** Evidence of the nitrate/nitrite on CAT, TAC, GSH and MDA in tissues of male rats with T2D.

Authors	Years	Treatment				Tissue	Effects			
		Type	Duration (Days)	Dose (mg/L)	Rout		CAT	TAC	GSH	MDA
Jeddi et al. [17]	2016	Nitrate	60	100	Drinking water	Heart	—	—	—	↑
Niu et al. [16]	2024	Nitrate	7	160	Drinking water	Skin	—	—	—	↓
Gheibi et al. [39]	2019	Nitrite	63	50	Drinking water	Liver	↑	↑	↑	↓
						SM	↑	↑	↑	↓
						eAT	↔	↑	↔	↔
Jeddi et al. [40]	2021	Nitrite	63	50	Drinking water	Kidney	↑	↔	↔	↓
Ghasemi et al. [3]	2023	Nitrite	60	50	Drinking water	Pancreatic islets	↑	—	—	—

Abbreviations: ↑, increase; ↓, decrease; ↔, no effect; CAT, catalase; eAT, epididymal adipose tissue; GSH reduced glutathione; MDA malondialdehyde; SM, soleus musles; T2D, Type 2 diabetes; TAC, total antioxidant capacity.

Although it cannot be inferred from the results of our study, the favourable effects of nitrate against T2D‐induced oxidative stress can be attributed to the conversion of nitrate to NO [16, 41, 42] and to the NO‐independent antioxidant effect of nitrate [14, 15, 43, 44]. Nitrate is reduced to nitrite and NO, which activates the nuclear factor erythroid 2‐related factor 2 (Nrf2), a key regulator of about 100 antioxidant genes [41, 42]. Nrf2 decreases oxidative markers, such as MDA and increases antioxidant enzymes, such as superoxide dismutase (SOD), in rats with T2D [16]. In support of both mechanisms underlying nitrate's antioxidant effects in rats with T2D, our results showed that nitrate increased NOx levels in insulin‐sensitive tissues, correlating with higher CAT, TAC and GSH levels and reduced MDA.

As a limitation, we did not determine the underlying mechanism of nitrate‐induced antioxidant effects, in particular its dependency to NO. As strengths, in this study, a low dose of nitrate was used for long‐term (100 mg/L for 6 months); this dose in rats (10.6 mg/kg for 6 months in rats [18]), which has antidiabetic effects [18, 45] and improves carbohydrate metabolism in eNOS knockout mice [46], is translated to 1.9 mg/kg for ~13 years in humans [47, 48], which is a safe dose for long‐term intervention [49]. In addition, we utilised the HFD‐STZ model of T2D, which closely mimics the pathophysiology of T2D in humans [23].

## 5 Conclusion

Nitrate reduced oxidative stress in insulin‐sensitive tissues of rats with T2D by increasing CAT, TAC and GSH, and decreasing MDA levels. Thus, the beneficial anti‐diabetic effects of nitrate are partly due to its ability to alleviate oxidative stress in these tissues. Nitrate is considered a biologically active compound with dual roles: functioning both as a nutrient and a signalling agent [14, 15], underscoring nitrate's complex biology beyond simply being a NO precursor. Considering the potential of nutrition‐based interventions involving nitrate, its supplementation might help reduce oxidative stress‐related complications in diabetic conditions in humans; a hypothesis that needs confirmation in human studies.

## Author Contributions

F.G., M.S., S.J. and A.G. designed the experiments; F.G., M.S. performed experiments and collected data; S.J. and A.G. discussed the results and strategy; A.G. Supervised, directed and managed the study; F.G., M.S., S.J. and A.G. drafted the article. F.G., M.S., S.J. and A.G., Final approved of the version to be published.

## Conflicts of Interest

The authors declare no conflicts of interest.

## Data Availability

Some or all datasets generated during and/or analysed during the current study are not publicly available but are available from the corresponding author on reasonable request.

## References

[1] International Diabetes Federation , IDF Diabetes Atlas teB (International Diabetes Federation, 2025), http://www.diabetesatlas.org.

[2] S. Banik and A. Ghosh , “The Association of Oxidative Stress Biomarkers With Type 2 Diabetes Mellitus: A Systematic Review and Meta‐Analysis,” Health Science Reports 4, no. 4 (2021): e389, 10.1002/hsr2.389. 34622023 PMC8485598

[3] A. Ghasemi , S. Gheibi , K. Kashfi , and S. Jeddi , “Anti‐Oxidant Effect of Nitrite in the Pancreatic Islets of Type 2 Diabetic Male Rats,” Iranian Journal of Basic Medical Sciences 26, no. 4 (2023): 420–428, 10.22038/ijbms.2023.68245.14900. 37009002 PMC10008387

[4] P. Drikvandi and S. Bahramikia , “Modulation of the Antioxidant Defense System in Liver, Kidney, and Pancreas Tissues of Alloxan‐Induced Diabetic Rats by Camphor,” Journal of Food Biochemistry 44, no. 12 (2020): e13527, 10.1111/jfbc.13527. 33084110

[5] S. Sánchez‐Duarte , E. Sánchez‐Duarte , L. A. Sánchez‐Briones , et al., “Apocynin Mitigates Diabetic Muscle Atrophy by Lowering Muscle Triglycerides and Oxidative Stress,” International Journal of Molecular Sciences 26, no. 12 (2025): 5636, 10.3390/ijms26125636. 40565097 PMC12193051

[6] A. Diniz and M. G. Alves , “Type 2 Diabetes Induces a Pro‐Oxidative Environment in Rat Epididymis by Disrupting SIRT1/PGC‐1α/SIRT3 Pathway,” International Journal of Molecular Sciences 23, no. 16 (2022): 8912, 10.3390/ijms23168912. 36012191 PMC9409047

[7] A. Caturano , M. D'Angelo , A. Mormone , et al., “Oxidative Stress in Type 2 Diabetes: Impacts From Pathogenesis to Lifestyle Modifications,” Current Issues in Molecular Biology 45, no. 8 (2023): 6651–6666, 10.3390/cimb45080420. 37623239 PMC10453126

[8] A. Ghasemi and R. Norouzirad , “Type 2 Diabetes: An Updated Overview,” Critical Reviews in Oncology 24, no. 3 (2019): 213–222, 10.1615/CritRevOncog.2019030976. 32422019

[9] L. D. Roberts , T. Ashmore , A. O. Kotwica , et al., “Inorganic Nitrate Promotes the Browning of White Adipose Tissue Through the Nitrate‐Nitrite‐Nitric Oxide Pathway,” Diabetes 64, no. 2 (2015): 471–484, 10.2337/db14-0496. 25249574 PMC4351918

[10] S. Gheibi , S. Jeddi , M. Carlström , H. Gholami , and A. Ghasemi , “Effects of Long‐Term Nitrate Supplementation on Carbohydrate Metabolism, Lipid Profiles, Oxidative Stress, and Inflammation in Male Obese Type 2 Diabetic Rats,” Nitric Oxide: Biology and Chemistry 75 (2018): 27–41, 10.1016/j.niox.2018.02.002. 29432804

[11] H. S. Brunetta , H. L. Petrick , I. Momken , et al., “Nitrate Consumption Preserves HFD‐Induced Skeletal Muscle Mitochondrial ADP Sensitivity and Lysine Acetylation: A Potential Role for SIRT1,” Redox Biology 52 (2022): 102307, 10.1016/j.redox.2022.102307. 35398714 PMC9006675

[12] G. J. DesOrmeaux , H. L. Petrick , H. S. Brunetta , and G. P. Holloway , “Independent of Mitochondrial Respiratory Function, Dietary Nitrate Attenuates HFD‐Induced Lipid Accumulation and Mitochondrial ROS Emission Within the Liver,” American Journal of Physiology. Endocrinology and Metabolism 321, no. 2 (2021): E217–e228, 10.1152/ajpendo.00610.2020. 34229472

[13] I. Cordero‐Herrera , M. Kozyra , Z. Zhuge , et al., “AMP‐Activated Protein Kinase Activation and NADPH Oxidase Inhibition by Inorganic Nitrate and Nitrite Prevent Liver Steatosis,” Proceedings of the National Academy of Sciences of the United States of America 116, no. 1 (2019): 217–226, 10.1073/pnas.1809406115. 30559212 PMC6320503

[14] X. Li , S. Wu , Z. Cao , et al., “Sialin2 Functions as a Mammalian Nitrate Sensor to Sustain Mitochondrial Homeostasis,” *bioRxiv*: The Preprint Server for Biology, (2025), 10.1101/2025.05.04.652104.

[15] X. Li , O. Jiang , Z. Cao , et al., “Sialin2 Senses Nitrate to Activate Endosomal PI3K‐AKT‐NOS Signaling,” *bioRxiv*: The Preprint Server for Biology, (2025), 10.1101/2025.05.04.652107.

[16] Q. Niu , D. Li , W. Guo , Z. Feng , Z. Han , and Y. Yang , “Dietary Nitrate Maintains Homeostasis of Oxidative Stress and Gut Microbiota to Promote Flap Survival in Type 2 Diabetes Mellitus Rats,” BMC Endocrine Disorders 24, no. 1 (2024): 184, 10.1186/s12902-024-01691-5. 39256735 PMC11386097

[17] S. Jeddi , S. Khalifi , M. Ghanbari , F. Bageripour , and A. Ghasemi , “Effects of Nitrate Intake on Myocardial Ischemia‐Reperfusion Injury in Diabetic Rats,” Arquivos Brasileiros de Cardiologia 107, no. 4 (2016): 339–347, 10.5935/abc.20160137. 27849257 PMC5102480

[18] V. Khorasani , S. Jeddi , P. Yaghmaei , M. Tohidi , and A. Ghasemi , “Effect of Long‐Term Sodium Nitrate Administration on Diabetes‐Induced Anemia and Glucose Homeostasis in Obese Type 2 Diabetic Male Rats,” Nitric Oxide: Biology and Chemistry 86 (2019): 21–30, 10.1016/j.niox.2019.02.003. 30772502

[19] N. Yousefzadeh , S. Jeddi , K. Kashfi , and A. Ghasemi , “Long‐Term Inorganic Nitrate Administration Protects Against Ovariectomy‐Induced Osteoporosis in Rats,” EXCLI Journal 21 (2022): 1151–1166, 10.17179/excli2022-5082. 36320805 PMC9618708

[20] J. Tannenbaum and B. T. Bennett , “Russell and Burch's 3Rs Then and Now: The Need for Clarity in Definition and Purpose,” Journal of the American Association for Laboratory Animal Science 54, no. 2 (2015): 120–132. 25836957 PMC4382615

[21] A. Ghasemi and S. Jeddi , “Streptozotocin as a Tool for Induction of Rat Models of Diabetes: A Practical Guide,” EXCLI Journal 22 (2023): 274–294, 10.17179/excli2022-5720. 36998708 PMC10043433

[22] M. M. Bradford , “A Rapid and Sensitive Method for the Quantitation of Microgram Quantities of Protein Utilizing the Principle of Protein‐Dye Binding,” Analytical Biochemistry 72 (1976): 248–254, 10.1016/0003-2697(76)90527-3. 942051

[23] S. Gheibi , K. Kashfi , and A. Ghasemi , “A Practical Guide for Induction of Type‐2 Diabetes in Rat: Incorporating a High‐Fat Diet and Streptozotocin,” Biomedicine & pharmacotherapy = Biomedecine & pharmacotherapie 95 (2017): 605–613, 10.1016/j.biopha.2017.08.098. 28881291

[24] M. H. Hadwan , M. J. Hussein , R. M. Mohammed , et al., “An Improved Method for Measuring Catalase Activity in Biological Samples,” Biology Methods & Protocols 9, no. 1 (2024): bpae015, 10.1093/biomethods/bpae015. 38524731 PMC10957919

[25] I. F. Benzie and J. J. Strain , “Ferric Reducing/Antioxidant Power Assay: Direct Measure of Total Antioxidant Activity of Biological Fluids and Modified Version for Simultaneous Measurement of Total Antioxidant Power and Ascorbic Acid Concentration,” Methods in Enzymology 299 (1999): 15–27, 10.1016/s0076-6879(99)99005-5. 9916193

[26] J. Sedlak and R. H. Lindsay , “Estimation of Total, Protein‐Bound, and Nonprotein Sulfhydryl Groups in Tissue With Ellman's Reagent,” Analytical Biochemistry 25 (1968): 192–205, 10.1016/0003-2697(68)90092-4. 4973948

[27] K. Satoh , “Serum Lipid Peroxide in Cerebrovascular Disorders Determined by a New Colorimetric Method,” Clinica Chimica Acta 90, no. 1 (1978): 37–43, 10.1016/0009-8981(78)90081-5. 719890

[28] K. M. Miranda , M. G. Espey , and D. A. Wink , “A Rapid, Simple Spectrophotometric Method for Simultaneous Detection of Nitrate and Nitrite,” Nitric Oxide: Biology and Chemistry 5, no. 1 (2001): 62–71, 10.1006/niox.2000.0319. 11178938

[29] S. Abdulmalek , A. Eldala , D. Awad , and M. Balbaa , “Ameliorative Effect of Curcumin and Zinc Oxide Nanoparticles on Multiple Mechanisms in Obese Rats With Induced Type 2 Diabetes,” Scientific Reports 11, no. 1 (2021): 20677, 10.1038/s41598-021-00108-w. 34667196 PMC8526574

[30] R. F. Abdel‐Rahman , S. M. Ezzat , H. A. Ogaly , et al., “Ficus Deltoidea Extract Down‐Regulates Protein Tyrosine Phosphatase 1B Expression in a Rat Model of Type 2 Diabetes Mellitus: A New Insight Into Its Antidiabetic Mechanism,” Journal of Nutritional Science 9 (2020): e2, 10.1017/jns.2019.40. 32042410 PMC6984126

[31] C. Mishra , M. A. Khalid , N. Fatima , et al., “Effects of Citral on Oxidative Stress and Hepatic Key Enzymes of Glucose Metabolism in Streptozotocin/High‐Fat‐Diet Induced Diabetic Dyslipidemic Rats,” Iranian Journal of Basic Medical Sciences 22, no. 1 (2019): 49–57, 10.22038/ijbms.2018.26889.6574. 30944708 PMC6437455

[32] K. Satyanarayana , K. Sravanthi , I. A. Shaker , R. Ponnulakshmi , and J. Selvaraj , “Role of Chrysin on Expression of Insulin Signaling Molecules,” Journal of Ayurveda and Integrative Medicine 6, no. 4 (2015): 248–258, 10.4103/0975-9476.157951. 26834424 PMC4719485

[33] K. Szkudelska , M. Okulicz , I. Hertig , and T. Szkudelski , “Resveratrol Ameliorates Inflammatory and Oxidative Stress in Type 2 Diabetic Goto‐Kakizaki Rats,” Biomedicine & pharmacotherapy = Biomedecine & pharmacotherapie 125 (2020): 110026, 10.1016/j.biopha.2020.110026. 32092822

[34] H. Yaribeygi , T. Sathyapalan , S. L. Atkin , and A. Sahebkar , “Molecular Mechanisms Linking Oxidative Stress and Diabetes Mellitus,” Oxidative Medicine and Cellular Longevity 2020, no. 1 (2020): 8609213, 10.1155/2020/8609213. 32215179 PMC7085395

[35] B. E. Davidson and F. J. Hird , “The Estimation of Glutathione in Rat Tissues. A Comparison of a New Spectrophotometric Method With the Glyoxalase Method,” Biochemical Journal 93, no. 2 (1964): 232–236, 10.1042/bj0930232. 5891310 PMC1206282

[36] P. Boriskin , A. Deviatkin , A. Nikitin , O. Pavlova , and A. Toropovskiy , “Relationship of Catalase Activity Distribution in Serum and Tissues of Small Experimental Animals,” IOP Conference Series: Earth and Environmental Science 403 (2019): 12113.

[37] M. Tiedge , S. Lortz , J. Drinkgern , and S. Lenzen , “Relation Between Antioxidant Enzyme Gene Expression and Antioxidative Defense Status of Insulin‐Producing Cells,” Diabetes 46, no. 11 (1997): 1733–1742, 10.2337/diab.46.11.1733. 9356019

[38] I. Carlberg and B. Mannervik , “Glutathione Reductase,” Methods in Enzymology 113 (1985): 484–490, 10.1016/s0076-6879(85)13062-4. 3003504

[39] S. Gheibi , S. Jeddi , M. Carlström , K. Kashfi , and A. Ghasemi , “Hydrogen Sulfide Potentiates the Favorable Metabolic Effects of Inorganic Nitrite in Type 2 Diabetic Rats,” Nitric Oxide: Biology and Chemistry 92 (2019): 60–72, 10.1016/j.niox.2019.08.006. 31479766

[40] S. Jeddi , S. Gheibi , K. Kashfi , and A. Ghasemi , “Sodium Hydrosulfide Has no Additive Effects on Nitrite‐Inhibited Renal Gluconeogenesis in Type 2 Diabetic Rats,” Life Sciences 283 (2021): 119870, 10.1016/j.lfs.2021.119870. 34352258

[41] J. A. David , W. J. Rifkin , P. S. Rabbani , and D. J. Ceradini , “The Nrf2/Keap1/ARE Pathway and Oxidative Stress as a Therapeutic Target in Type II Diabetes Mellitus,” Journal of Diabetes Research 2017 (2017): 4826724, 10.1155/2017/4826724. 28913364 PMC5585663

[42] S. Baumel‐Alterzon , L. S. Katz , G. Brill , A. Garcia‐Ocaña , and D. K. Scott , “Nrf2: The Master and Captain of Beta Cell Fate,” Trends in Endocrinology and Metabolism 32, no. 1 (2021): 7–19, 10.1016/j.tem.2020.11.002. 33243626 PMC7746592

[43] S. Shiva , “Nitrite: A Physiological Store of Nitric Oxide and Modulator of Mitochondrial Function,” Redox Biology 1, no. 1 (2013): 40–44, 10.1016/j.redox.2012.11.005. 23710434 PMC3661298

[44] P. Waltz , D. Escobar , A. M. Botero , and B. S. Zuckerbraun , “Nitrate/Nitrite as Critical Mediators to Limit Oxidative Injury and Inflammation,” Antioxidants & Redox Signaling 23, no. 4 (2015): 328–339, 10.1089/ars.2015.6256. 26140517 PMC4692126

[45] M. Shokri , S. Jeddi , H. Faridnouri , V. Khorasani , K. Kashfi , and A. Ghasemi , “Effect of Nitrate on Gene and Protein Expression of Nitric Oxide Synthase Enzymes in Insulin‐Sensitive Tissues of Type 2 Diabetic Male Rats,” Endocrine, Metabolic & Immune Disorders Drug Targets 21, no. 12 (2021): 2220–2230, 10.2174/1871530321666210622155649. 34161215

[46] M. Carlstrom , F. J. Larsen , T. Nystrom , et al., “Dietary Inorganic Nitrate Reverses Features of Metabolic Syndrome in Endothelial Nitric Oxide Synthase‐Deficient Mice,” Proceedings of the National Academy of Sciences of the United States of America 107, no. 41 (2010): 17716–17720, 10.1073/pnas.1008872107. 20876122 PMC2955084

[47] R. Quinn , “Comparing Rat's to Human's Age: How Old Is My Rat in People Years?,” Nutrition 21, no. 6 (2005): 775–777, 10.1016/j.nut.2005.04.002. 15925305

[48] S. Reagan‐Shaw , M. Nihal , and N. Ahmad , “Dose Translation From Animal to Human Studies Revisited,” FASEB Journal: Official Publication of the Federation of American Societies for Experimental Biology 22, no. 3 (2008): 659–661, 10.1096/fj.07-9574LSF. 17942826

[49] N. S. Bryan and J. L. Ivy , “Inorganic Nitrite and Nitrate: Evidence to Support Consideration as Dietary Nutrients,” Nutrition Research (New York, NY) 35, no. 8 (2015): 643–654, 10.1016/j.nutres.2015.06.001. 26189149

